# Artificial intelligence and sexual health in the USA

**DOI:** 10.1016/S2589-7500(21)00117-5

**Published:** 2021-08

**Authors:** Sean D Young, Jeffrey S Crowley, Sten H Vermund

**Affiliations:** Department of Emergency Medicine, and Department of Informatics, Bren School of Information and Computer Sciences, University of California, Irvine, CA 92868, USA; O’Neill Institute for National and Global Health Law, Georgetown University, Washington, DC, USA; School of Public Health, Yale University, New Haven, CT, USA

The US Centers for Disease Control and Prevention (CDC) estimated that one in five people in the USA had a sexually transmitted infection (STI) in 2018.^1^ Although widespread and a substantial threat to the health of the US population, STIs have not received the attention required to prevent continued transmission.^2^ Despite rising rates of STIs over the past decade, CDC funding for STI prevention has not changed, and, when accounting for inflation, has reduced by 40% between 2003 and 2020.^2^ We are members of the Committee on the Prevention and Control of Sexually Transmitted Infections in the US National Academies of Sciences, Engineering, and Medicine (NASEM). The committee was commissioned by the CDC to review current public health strategies and provide recommendations on future programmes, policy, and research.^2^ In March, 2021, the committee released a consensus report with a central recommendation of embracing innovation as a means to improve sexual health. One innovation is the use of artificial intelligence (AI) to enhance STI prevention and control. Because of the rapid growth in AI applications in health, we believe that AI can be used as part of a solution to address STI epidemics.

AI can improve STI surveillance and interventions. For example, STI researchers have already shown that AI can predict county-level rates of syphilis by analysing publicly available social media data of people’s sexual attitudes and behaviours associated with syphilis.^3^ Although extremely laborious through traditional surveillance methods, AI allows this analysis to be completed in seconds. Here, we track the four major action areas of the consensus report to describe ways that AI can be integrated into an effective STI response.

AI can foster an improved approach to sexual health, both for individuals and society. A change in sexual health approach requires a fundamental change in the attitudes and language used by providers, individuals, and policy makers. AI can analyse massive amounts of publicly available online data (eg, social media and online forums) to learn about trends in stigmatising language and inform efforts to prevent stigma around STI prevention and care. Similar to the methods being used to identify and flag misinformation, AI could identify and flag (eg, notify users, parents, and other key stakeholders) when STI-related misinformation is detected.

Responsibility for STI prevention and control needs to be expanded beyond STI clinics to include primary care, speciality care, and community stakeholders. Proof-of-concept studies are already using AI as a tool to analyse the electronic health records of people with HIV and to identify patients who might benefit from pre-exposure prophylaxis.^4^ Similarly, AI might be used to analyse patterns among electronic health records and insurance claims data to assess risk factors for STIs and assess how they might vary among demographic groups to tailor intervention efforts. These data could inform key stakeholders (eg, parents, religious leaders, and racial or minority ethnic communities) about where, when, and how to best engage their communities with appropriate sexual health resources. AI models suggesting trends in the use of services for STI care could inform public health bodies of the resources needed for STI prevention and treatment, including medications.

Responding to STIs has been, and should continue to be, grounded in public health approaches. CDC leadership is crucial, but essential public health measures, such as surveillance and distribution of funds for STI prevention and treatment, are done by states and local governments. AI tools can help to address case-based reporting delays and other limitations of current STI data collection. Optimal STI surveillance relies on timely and accurate data, yet surveillance data are often delayed or unavailable. AI could analyse patterns among electronic health records and claims data and combine it with analysis of near real-time social media, dating app, and mobility data. The insights from these analyses—especially analyses based on near real-time data—are shown to help predict STI cases and are available much quicker than traditional surveillance efforts.^5^

STIs disproportionately affect stigmatised groups, such as sexual minority and gender diverse groups, and minority ethnic communities.^6,7^ The improving technology and data footprints have tremendous potential to address the structural drivers of STIs. Immersive virtual reality applications might use AI to increase understanding of experiences and the stigma of people who have contracted STIs. AI-informed virtual reality (ie, AI to learn from the experiences of each virtual reality user) has the potential to create an engaging and empathy-building educational environment.^8^ Just as AI-informed virtual reality therapist applications have been studied for use in mental health treatment settings,^9^ similar applications might be developed and studied for providing STI education, prevention, and treatment resources. Many of the communities face structural barriers to accessing accurate information, STI prevention services, and STI care services, and AI offers new possibilities for providing tailored resources that can educate communities and offer new pathways for accessing resources. Social media and online community data might be analysed to assess the effect of online racism (eg, racist comments) on STI rates by region. In line with the belief that reducing racism and discrimination would reduce STI transmission,^2^ AI might also be used to improve anti-racism interventions, such as identifying racist and sexually offensive content online.

Reports on the biases from AI tools (eg, racial bias and discrimination) warrant discussion.^10^ Because AI models are, at least initially, developed by humans, they have similar biases as humans. Machines, similar to humans, are likely to be racially biased if they have little education and experience. Just as racial integration and positive exposure to a diverse range of racial and ethnic groups help to reduce racism, providing a machine with large amounts of data from people from diverse backgrounds can reduce biases and increase accurate and equitable decision making. However, there might still be deep-rooted biases in this data and a prominent focus on equity by diverse research teams will be essential to achieving more equitable outcomes.

Effective implementation of AI requires extensive participation by stakeholders in the design, implementation, and monitoring of tools. Care also should be taken to ensure that STI interventions are ethical. US President Joe Biden has already suggested that AI will be crucial in his governing agenda, reinforcing the possibility that infrastructure for AI in sexual health will be available and can be provided equitably. The USA can do more to prevent STIs and better control their spread and harmful sequelae. AI solutions can be part of the answer.
